# Contextual Acceptance of COVID-19 Mitigation Mobile Apps in the United States: Mixed Methods Survey Study on Postpandemic Data Privacy

**DOI:** 10.2196/57309

**Published:** 2024-08-29

**Authors:** Yuanyuan Feng, Brad Stenger, Shikun Zhang

**Affiliations:** 1 Department of Computer Science University of Vermont Burlington, VT United States; 2 School of Computer Science Carnegie Mellon University Pittsburgh, PA United States

**Keywords:** data privacy, health privacy, COVID-19, mobile apps, contextual integrity, respiratory, infectious, pulmonary, pandemic, mobile app, app, apps, digital health, digital technology, digital intervention, digital interventions, smartphone, smartphones, mobile phone

## Abstract

**Background:**

The COVID-19 pandemic gave rise to countless user-facing mobile apps to help fight the pandemic (“COVID-19 mitigation apps”). These apps have been at the center of data privacy discussions because they collect, use, and even retain sensitive personal data from their users (eg, medical records and location data). The US government ended its COVID-19 emergency declaration in May 2023, marking a unique time to comprehensively investigate how data privacy impacted people’s acceptance of various COVID-19 mitigation apps deployed throughout the pandemic.

**Objective:**

This research aims to provide insights into health data privacy regarding COVID-19 mitigation apps and policy recommendations for future deployment of public health mobile apps through the lens of data privacy. This research explores people’s contextual acceptance of different types of COVID-19 mitigation apps by applying the privacy framework of contextual integrity. Specifically, this research seeks to identify the factors that impact people’s acceptance of data sharing and data retention practices in various social contexts.

**Methods:**

A mixed methods web-based survey study was conducted by recruiting a simple US representative sample (N=674) on Prolific in February 2023. The survey includes a total of 60 vignette scenarios representing realistic social contexts that COVID-19 mitigation apps could be used. Each survey respondent answered questions about their acceptance of 10 randomly selected scenarios. Three contextual integrity parameters (attribute, recipient, and transmission principle) and respondents’ basic demographics are controlled as independent variables. Regression analysis was performed to determine the factors impacting people’s acceptance of initial data sharing and data retention practices via these apps. Qualitative data from the survey were analyzed to support the statistical results.

**Results:**

Many contextual integrity parameter values, pairwise combinations of contextual integrity parameter values, and some demographic features of respondents have a significant impact on their acceptance of using COVID-19 mitigation apps in various social contexts. Respondents’ acceptance of data retention practices diverged from their acceptance of initial data sharing practices in some scenarios.

**Conclusions:**

This study showed that people’s acceptance of using various COVID-19 mitigation apps depends on specific social contexts, including the type of data (attribute), the recipients of the data (recipient), and the purpose of data use (transmission principle). Such acceptance may differ between the initial data sharing and data retention practices, even in the same context. Study findings generated rich implications for future pandemic mitigation apps and the broader public health mobile apps regarding data privacy and deployment considerations.

## Introduction

### Background

To combat the COVID-19 pandemic, countless user-facing mobile apps (“COVID-19 mitigation apps”) have been developed and deployed around the world [1,2]. These apps provide various functionalities, including contact tracing [3], symptom self-checking [4], test result reporting (eg, iHealth COVID-19 Test [iHealth Labs Inc]), and proof of vaccination (eg, Excelsior [New York State]). However, low adoption rates often undermine the real-world impact of these apps [5], partially caused by people’s data privacy concerns around how these apps handle various sensitive personal data, including medical records and phone-based location data [6,7].

Public health priorities have changed throughout the COVID-19 pandemic [8], resulting in evolving needs for pandemic mitigation apps. Some apps deployed early in the pandemic with narrow functionality (eg, symptom self-checking) became obsolete, while other apps are likely to remain useful after the pandemic (eg, test result reporting). The United States ended the COVID Public Health Emergency on May 11, 2023 [9]. It is time to comprehensively examine user privacy across different types of COVID-19 mitigation apps developed in this pandemic. Specifically, we aim to investigate 3 types of COVID-19 mitigation apps with broad utility in future public health crises, namely, contact tracing, test result reporting, and proof of vaccination.

We conducted a vignette survey study that applied the privacy framework of contextual integrity [10] to examine people’s acceptance of how COVID-19 mitigation apps could handle their personal health data in various social contexts. This research informs the design and deployment of future public health apps and relevant mandates around these apps from a privacy-centric perspective. Insights from this research could encourage the adoption of future public health apps by addressing people’s data privacy concerns in diverse social contexts.

### Prior Work: Data Privacy and the Adoption of COVID-19 Mitigation Apps

Research examined the adoption of mobile technologies for pandemic mitigation worldwide, which primarily focused on contact tracing apps [3,11-14]. Generally, adoption rates are high for COVID-19 mitigation apps backed by government mandates, such as Aarogya Setu (National Informatics Centre, Government of India) and HaMagen (Health Ministry, Israel), but low for apps relying on users’ voluntary installation (eg, COVID-19 exposure alerts in the United States). A multicountry study shows that people in collective cultures are more likely to adopt contact tracing apps than those in strong individualistic cultures [15].

Among all the factors impacting user adoption of COVID-19 mitigation apps [13,14,16-18], data privacy stood out as a major concern [3,6,7,15,19,20]. Recent qualitative and mixed methods studies also unpacked individual users’ complex decision-making process, including data privacy considerations when adopting COVID-19 mitigation apps [21,22].

However, most studies heavily focused on initial adoption but overlooked the continued use or future use of these apps after the pandemic. In addition, many studies only examined a single app type [3,6,7,21,22], neglecting the fact that multiple types of apps can be used together to fight the pandemic. Our study bridges these gaps by examining 3 major types of COVID-19 mitigation apps with long-term utility in future public health crises.

Another gap in the literature was the retention and reuse of personal data collected by COVID-19 mitigation apps. Data retention is central to privacy law discussion [23,24] and affects users’ willingness to share personal information [25]. Currently, there is no clear guidance on how various data collected by these apps should be retained, while controversial reuse of such data by law enforcement has been reported [26,27]. Misuse of retained data from COVID-19 mitigation apps can have dire privacy consequences. Our study provides a new understanding of users’ attitudes toward COVID-19 mitigation apps’ data retention.

### Theory: The Privacy Framework of Contextual Integrity

People’s data privacy attitudes toward computing technologies differ in various contexts [28-30], including who collects the data, the purpose of the data collection, and the specific ways the collected data will be used, processed, or shared. Hargittai et al [18] found that US participants’ willingness to adopt contact tracing apps differs by the providers of the apps; Zhang et al [31] revealed that people’s acceptance of vaccination certificates varies in diverse real-world scenarios. However, traditional privacy theories (eg, public or private dichotomy) fail to consider the contexts for computing technologies’ complex data practices [32,33].

Privacy as contextual integrity, conceptualized by Nissenbaum [10], is a rising privacy framework to examine computing technologies’ highly complex data practices. Contextual integrity defines privacy as the appropriate flow of information that follows established information norms in a particular society or culture. According to contextual integrity, information norms can be captured in 5 contextual integrity parameters: sender (who sends the data), recipient (who receives the data), subject (whose data), attribute (type of information), and transmission principle (TP thereafter, conditions of the flow). Empirically, we can observe information norms from people’s privacy attitudes toward different information flows exhibited in various data practices.

Compared to prior privacy theories, the contextual integrity framework is advantageous in identifying potential privacy violations typically when 1 or more of the parameters deviate from an established information norm. For example, it might be considered appropriate for a store owner (recipient) to collect vaccination information (attribute) from a customer (sender and subject) before letting them into the store (TP). However, if the business owner were to use this information for advertising purposes or keep the data indefinitely, the resulting flows, with different TPs, would deviate from the established information norms.

Built upon insights from prior studies leveraging contextual integrity to examine the information flows for public health apps [15,31,34], this study rigorously applies the contextual integrity framework to examine people’s acceptance of COVID-19 mitigation apps in highly diverse contexts.

### Research Questions and Hypotheses

We aim to answer 2 research questions (RQs) and 4 specific hypotheses by considering applicable contextual integrity parameters and respondents’ demographics.

- RQ1: What factors impact people’s acceptance of sharing data via COVID-19 mitigation apps?
  Null hypothesis 1.1 (NH1.1): There is no difference in people’s acceptance of sharing health data via COVID-19 mitigation apps, even if contextual integrity parameters are different.
  Null hypothesis 1.2 (NH1.2): There is no difference in people’s acceptance of sharing data via COVID-19 mitigation apps, even if they have different demographic backgrounds.
- RQ2: What factors impact people’s acceptance of their data being retained via COVID-19 mitigation apps?
  Null hypothesis 2.1 (NH2.1): There is no difference in people’s acceptance of their data being retained via COVID-19 mitigation apps, even if contextual integrity parameters are different.
  Null hypothesis 2.2 (NH2.1): There is no difference in people’s acceptance of their data being retained via COVID-19 mitigation apps, even if they have different demographic backgrounds.

## Methods

### Variables and Measurements

We designed a contextual integrity–based survey instrument according to our RQ and hypotheses. The outcome variable is “perceived acceptance,” a proxy to measure people’s privacy attitudes toward COVID-19 mitigation apps. For RQ1, we use a 5-point Likert scale question to capture different acceptance levels because people’s privacy preferences are often nonbinary [35]. For RQ2, we chose not to specify a retention time due to the evolving COVID-19 guidelines (eg, how long a person should isolate after getting COVID-19). Instead, we measure acceptance using a 3-level categorical variable to capture necessary nuances.

The independent variables are applicable contextual integrity parameters and some demographic features self-reported by respondents. According to contextual integrity, it is crucial to include all 5 contextual integrity parameters to comprehensively evaluate the appropriateness of information flow, so we follow a standard contextual integrity–based template shown in Table 1 to construct survey questions including all parameters. We stabilized 2 contextual integrity parameters ([sender] and [subject] are “you” and “your,” which refer to the survey respondent) and varied the values of 3 contextual integrity parameters ([attribute], [recipient], and [TP]) as our independent variables.

**Table 1:** Example survey questions for 1 vignette scenario.

Question numbers	Example survey questions
Contextual integrity–based template	Would it be acceptable or unacceptable for [Sender] to share [Subject’s] [Attribute] with [Recipient] for [Transmission Principle (TP)]?
Question 1	Via a smartphone mobile app, would it be acceptable or unacceptable for *you* [Sender] to share *your* [Subject’s] *recent COVID test result* [Attribute] with *your employer* [Recipient] to work in person [TP]?
Question 1 follow-up	Please briefly explain your choice to the previous question (1-2 sentences).
Question 2	In the same scenario above, if *you* [Sender] shared *your* [Subject] *recent COVID test result* [Attribute] via a smartphone mobile app, would it be acceptable or unacceptable for *your employer* [Recipient] to *keep your data* [TP]?
Question 2 follow-up	Please briefly explain your choice to the previous question (1-2 sentences).

### Survey Design

We designed a mixed methods survey using vignette scenarios, which are effective to study people’s beliefs [36] and conduct survey-based experiments [37]. Vignette scenarios are widely used in privacy research to capture people’s contextual privacy attitudes toward digital technologies [30,38-41], enabling us to examine the diverse use cases of COVID-19 mitigation apps in real-world contexts. To craft realistic vignette scenarios, we conducted a technology review of COVID-19 mitigation apps available in the United States and gathered news articles about other COVID-19 migration apps deployed around the world. We used the scenarios from our recently published research [31] and slightly modified them to match the scope of this study. Next, we chose a subset of scenarios that would apply to contact tracing, proof of vaccination, and test result reporting apps and then finalized all the values of the 3 contextual integrity parameters, as shown in Textbox 1.

We then applied a full factorial design across the 3 contextual integrity parameters in Textbox 1 to generate 60 distinct scenarios. Each scenario contains 2 main questions corresponding to the 2 RQs: the first question presents the scenario to gauge acceptance and the second question examines the acceptance toward data retention by the recipient in the scenario. Note that contextual integrity considers data retention as a type of TP, so both questions conform to the standard contextual integrity–based template.

**All contextual integrity parameter values in our survey (text in italics format indicates baseline values in our models).**

Attribute: (1) *Recent COVID-19 test results*, (2) up-to-date COVID-19 vaccination records, and (3) COVID-19 exposure status from phone-based contact tracing.

Recipient: (1) Employers, (2) *essential stores (grocery stores, etc)*, (3) nonessential stores (department stores, etc), (4) dine-in locations (restaurants, bars, etc), (5) large public venues (stadium, music festival, etc), (6) health care providers (hospitals, clinics, specialists, etc), (7) airlines operating international flights, (8) airlines operating domestic flights, (9) domestic long-distance transportation operators (trains, Greyhound, Megabus, etc), and (10) local public transit operators (subways, metros, commuter trains, buses, etc).

Transmission principle: (1) *Public health purpose* and (2) scenario-specific purpose (eg, gain access to [location] or use [service]).

### Survey Questionnaire

The survey questionnaire began with a consent page, which confirmed participant eligibility and obtained informed consent. Then, the questionnaire displayed 2 information pages, including plain language definitions of COVID-19–related terminology and examples of some COVID-19 mitigation apps deployed in the United States. At the end of the information pages, we asked respondents to confirm their understanding of the information. Failure to confirm did not disqualify respondents, but their responses were excluded from data analysis.

Afterward, the questionnaire displayed 10 randomly selected vignette scenarios to limit the study time to around 10 minutes to minimize survey fatigue [42]. As shown in Table 1, each scenario has 2 contextual integrity–based questions. In 2 of 10 vignette scenarios, we inserted 2 follow-up free-text questions to gather additional qualitative data to understand respondents’ rationale. Finally, the questionnaire ended with a set of culturally responsible demographic questions for the US population and additional background questions on their COVID-19 experience.

For attention check, we avoided conventional attention check questions that are irrelevant to the survey because they are ineffective and may undermine researcher-participant trust [43]. Instead, we used multiple metrics as attention and accuracy checks, including the time spent on each scenario and the quality of free-text responses.

### Ethical Considerations

This survey study was approved by the institutional review board at the University of Vermont under the exempt category, where the written consent form is waived. We obtained informed consent through a consent page displayed at the beginning of the survey. All survey responses were deidentified by the Prolific platform Academic Ltd and used for data collection. Except obvious survey abuse, all respondents were each compensated US $2 via the Prolific platform (average compensation rate=US $10.43 per hour). To ensure research credibility, we preregistered this study in the Open Science Framework (Center for Open Science, Inc) before data collection, which is available to the public [44].

### Recruitment and Data Collection

We implemented the survey questionnaire in the Qualtrics XM platform (Qualtrics, LLC) for web-based distribution and chose the Prolific platform (Prolific Academic Ltd) to recruit respondents to maximize data collection speed and quality. We ran 3 small pilot surveys in December 2022 and January 2023, which led to minor refinements of the questionnaire. Next, we distributed the finalized survey in February 2023 and recruited a US representative sample via Prolific [45]. We received 694 completed survey responses and excluded 2 responses due to obvious survey abuse. The remaining 692 survey respondents were each compensated US $2 according to our study protocol.

We performed a 2-step data cleaning procedure using a customized Python script, followed by manual inspection. This led to the exclusion of 18 responses due to the lack of substance in the free-text answers (n=12) and the nonsensical content of the free-text answers (n=6). We included the remaining 674 survey responses in the final analysis.

### Statistical Analysis

#### Overview

Because our outcome variables for both RQs are ordinal data, multilevel ordered logistic regression is suitable to examine the effects of multiple predators. We used the cumulative link mixed models (clmm) in the ordinal package in R (R Foundation for Statistical Computing) [46] with random effects to account for repeated measures in the survey.

#### Predictors

We selected the most promising variables as predictors to construct our models, as follows:

#### Main Effect Predictors

We include all values of attribute, recipient, and TP, as well as 6 demographic variables (age, gender, political leaning, living areas, education, and income), and 4 COVID-19 experience questions (installation of contact tracing apps, being tested positive for COVID-19, being vaccinated against COVID-19, and knowing someone who got seriously ill or passed away from COVID-19) as main effect predictors.

#### Effect Modifiers

We account for the interactions among contextual integrity parameters by including effect modifications to the estimates of individual main effects. We include all pairwise combinations of the 3 contextual integrity parameters (ie, attribute*recipient, recipient*TP, and TP*attribute) as effect modifiers in our models. These effect modifiers enable us to interpret special cases when 2 specific contextual integrity parameters are present.

#### Predictor Baselines

For each predictor and effect modifier, we set the baseline value to the most common option according to the US population’s familiarity. For example, we chose COVID-19 test results as the baseline for attribute because app-based contact tracing and digital vaccination records were less commonly adopted in the United States. The baseline values for contextual integrity parameters are set in italics format in Table 1.

#### Final Models

We constructed 2 ordered logistic regression models for 2 RQs. Our models are inherently complex due to the large number of main effect predictors and effect modifiers. To ensure model convergence, we combined data categories in some demographic variables (eg, age groups and political leaning) to reduce model complexity. We also converted “not sure” and “prefer not to answer” responses to the “not available (N/A)” placeholder value to maximize model convergence. Our models used the “ucminf” optimizer in place of the package default optimizer “nlminb.” Both are commonly used optimizer algorithms to maximize the marginal likelihood function.

### Results Reporting and Interpretation

We followed the best practices outlined in Taylor et al [47] to report and interpret our model results. For statistically significant predictors (*P*<.05), we reported the odds ratios (ORs) of the effect, where OR is the natural exponent of the model estimate (β) for a predictor. We also report the 95% CIs of ORs.

For main effect interpretation, if a predictor value has an OR of 2, it means respondents are twice as likely to accept the scenario when compared to the baseline value of the predictor. To interpret the interactions among 2 contextual integrity parameters, we must calculate the marginal ORs of the main effect predictors and effect modifiers using the formula: e^(β_main_effect_predictor_+β_effect_modifier_). For example, if the estimate for a main effect predictor value is 2 (OR 7.39), and the estimate of the effect modifier containing a second predictor value is 0.5, then the marginal OR is e^(2+0.5)=12.18. This means the presence of the second predictor value increased the OR of the main effect predictor value from 7.39 to 12.18 compared to the main effect predictor baseline value. We also calculated the marginal 95% CIs for these interactions.

Each participant answered free-text follow-up questions for 2 randomly selected scenarios. After excluding low-quality free-text responses, we collected 2679 responses in total: 1340 for question 1 (44.7 per scenario) and 1339 for question 2 (44.6 per scenario). We performed a simplified qualitative analysis to synthesize notable themes from these responses and then selected representative quotes to further explain or support our statistical results.

## Results

### Respondents and Descriptive Statistics

**Figure 1:**
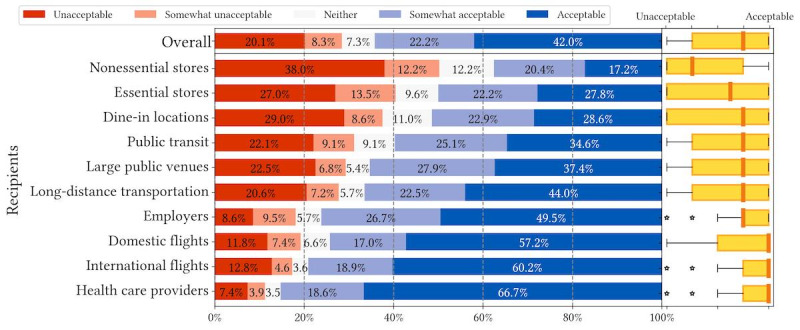
Descriptive statistics of question 1 responses: acceptance of sharing recent COVID-19 test results in different vignette scenarios organized by recipients.

**Figure 2:**
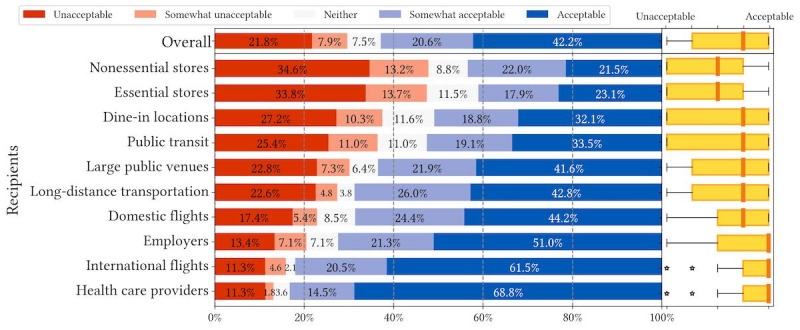
Descriptive statistics of question 1 responses: acceptance of sharing up-to-date COVID-19 vaccination records in different vignette scenarios organized by recipients.

**Figure 3:**
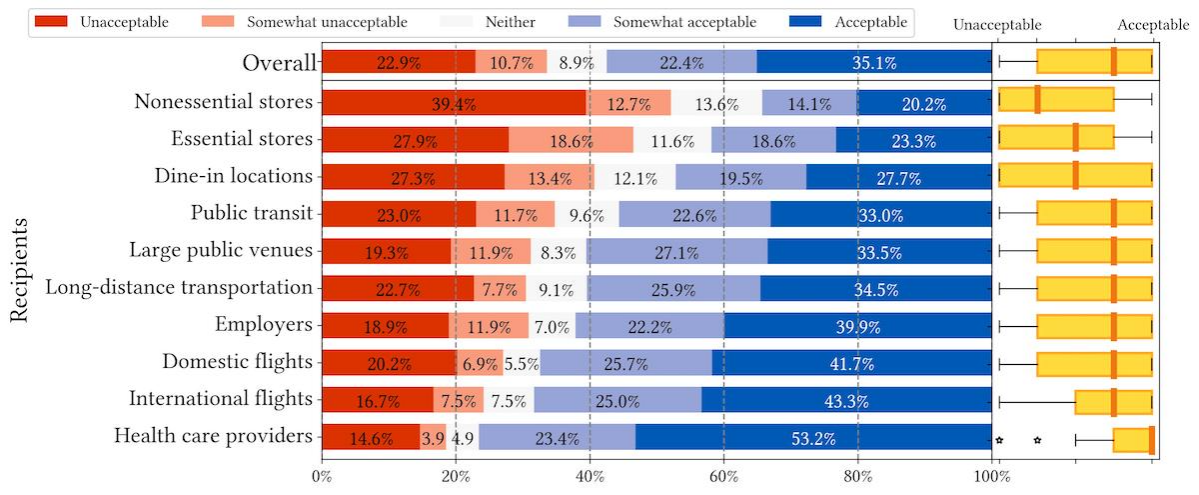
Descriptive statistics of question 1 responses: acceptance of sharing COVID-19 exposure status from phone-based contact tracing in different vignette scenarios organized by recipients.

**Figure 4:**
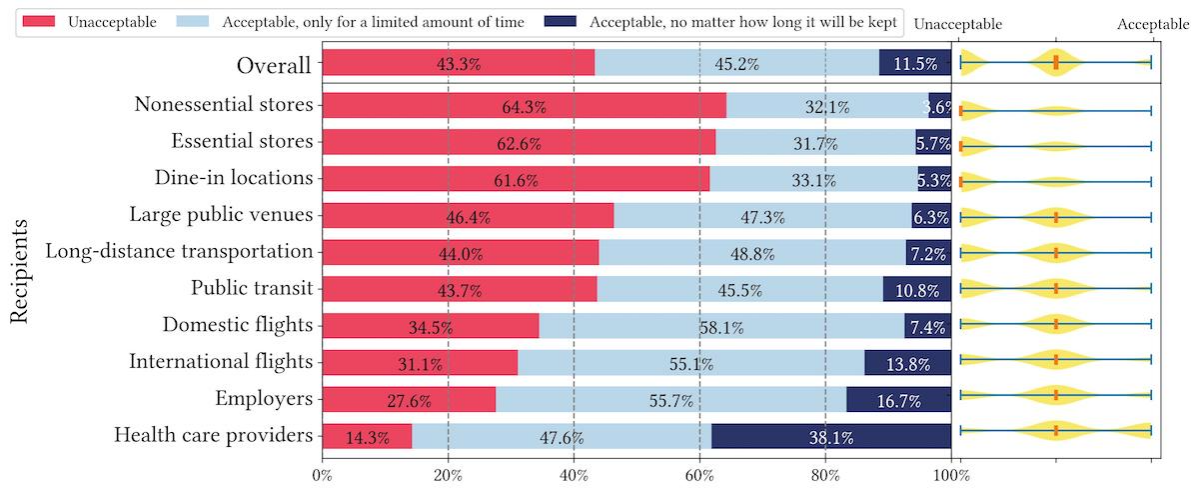
Descriptive statistics of question 2 responses: acceptance of recent COVID-19 test results being retained in different vignette scenarios organized by recipients.

**Figure 5:**
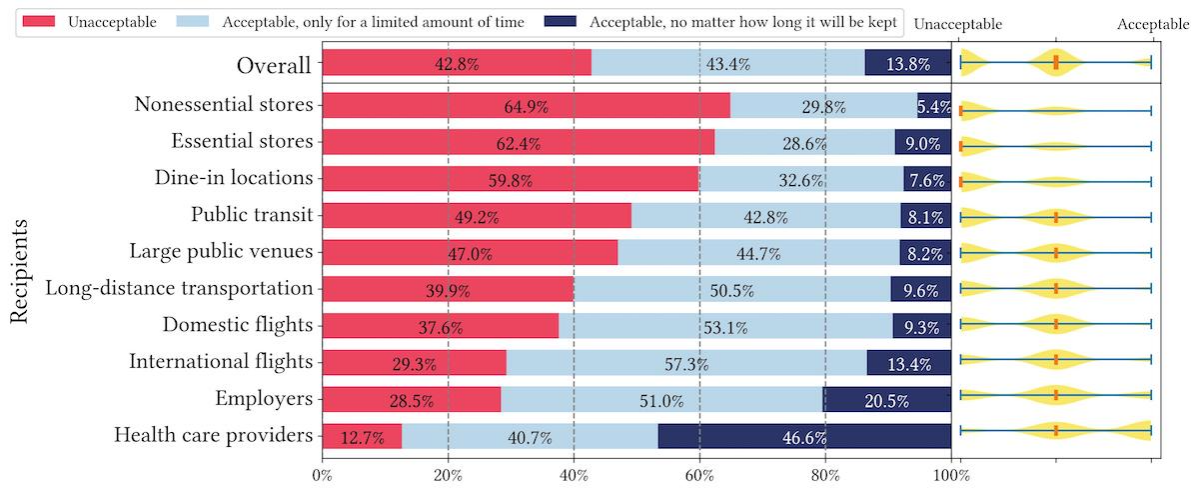
Descriptive statistics of question 2 responses: acceptance of up-to-date COVID-19 vaccination records being retained in different vignette scenarios organized by recipients.

**Figure 6:**
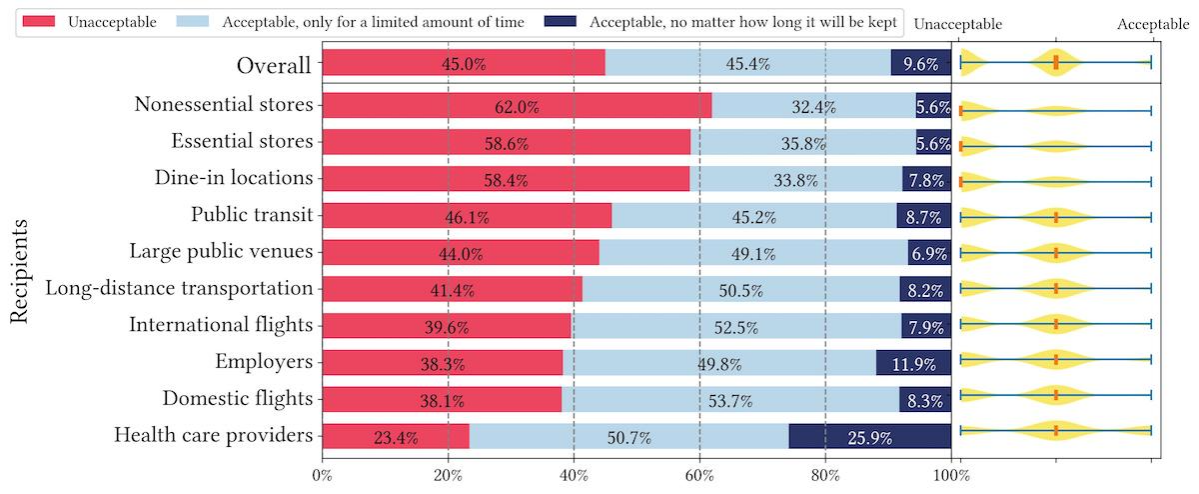
Descriptive statistics of question 2 responses: acceptance of COVID-19 contact tracing exposure status being retained in different vignette scenarios organized by recipients.

We recruited a simple US representative sample (N=674) via Prolific that resembles US Census data across age, gender, and race or ethnicity (Multimedia Appendix 1). We present the overall descriptive statistics of question 1 and question 2 responses in Figures 1-6.

Figures 1-3 depict respondents’ acceptance levels for sharing their data via COVID-19 mitigation apps in all the vignette scenarios. Generally, data sharing is mostly acceptable, as respondents found data sharing “acceptable” or “somewhat acceptable” in 61.5% (4147/6740) of all scenarios (referred to as “overall acceptance” thereafter). Across all attributes, sharing is most acceptable when recipients are health care providers (538/657, 81.9%), employers (484/692, 69.9%), and airlines operating international flights (515/675, 76.3%). Regarding attributes, respondents were less comfortable sharing COVID-19 exposure status from contact tracing (1284/2233, 57.5% overall acceptance) than sharing vaccination records (1435/2283, 62.9% overall acceptance) or test results (1428/2224, 64.2% overall acceptance).

Figures 4-6 show respondents’ acceptance levels for their health data being retained by recipients in all vignette scenarios. Across all attributes and recipients, considerable numbers of respondents felt data retention was “unacceptable” (2947/6740, 43.7% of all scenarios) or “acceptable, only for a limited amount of time” (3010/6740, 44.7% of all scenarios). Only in 11.6% (783/6740) of all scenarios, respondents reported recipients retaining their health data as “acceptable, no matter how long it will be kept” (or acceptable without a time limit). Across all recipients, health care providers retaining health data is most accepted, but only in 37.1% (244/657) of health care provider scenarios, respondents felt data retention was acceptable without a time limit. Similarly, small percentages of respondents found data retention without a time limit acceptable across all attributes: 9.6% (214/2233) of scenarios for contact tracing exposure status, 13.8% (314/2283) of scenarios for vaccination records, and 11.4% (255/2224) of scenarios for test results. The only outliner is health care providers retaining vaccination records, where 46.6% (103/221) responses to this scenario were acceptable without a time limit. Overall, question 2 results are largely consistent with question 1 results in terms of recipients, while retention time differentiates respondents’ acceptance levels drastically.

### RQ1: Acceptance of Data Sharing Through COVID-19 Mitigation Apps

#### Contextual Integrity Parameters

As shown in Table 2, recipient is the most powerful contextual integrity parameter to predict respondents’ acceptance levels toward COVID-19 mitigation apps. The recipients with the largest effects were health care providers (OR 146.36, 95% CI 78.06-274.42; *P*<.001), employers (OR 10.94, 95% CI 6.37-18.78; *P*<.001), and airlines operating domestic (OR 11.34, 95% CI 6.53-19.69; *P*<.001) and international flights (OR 16.03, 95% CI 8.88-28.93; *P*<.001), where respondents were more willing to use mobile apps to share their personal health information with them compared to the baseline recipient of essential stores. Respondents’ free-text responses revealed that the most accepted recipients are in scenarios that could critically impact the health of others and themselves. For example, respondents commented that sharing data with health providers “is necessary to keep track of the virus ... also necessary to keep myself and others healthy,” sharing data with airlines “would help keep travelers safe,” and sharing data with employers “is important for my coworkers to feel safe when I come to work.”

Additionally, large public venues (OR 4.13, 95% CI 2.45-6.97; *P*<.001), local public transit operators (OR 3.12, 95% CI 1.84-5.27; *P*<.001), and long-distance transportation operators (OR 5.87, 95% CI 3.42-10.09; *P*<.001) were also more tolerated than the baseline. In contrast, sharing data via mobile apps with nonessential stores (OR 0.35, 95% CI 0.21-0.59; *P*<.001) was less accepted than the baseline. Respondents’ comments explained the reasons: “Nonessential stores do not need this data.” “That’s too drastic. You can’t deny people access to food and supplies.” Only dine-in locations had no statistically significant effect.

However, no attribute or TP value has a significant effect. Respondents did not report significant differences in acceptance levels toward sharing contact tracing exposure status or proof of vaccination from test results. Similarly, scenario-specific purposes are not statistically different from generic public health purposes. Note that we group all scenario-specific purposes into 1 TP value to ensure model convergence, which means our model may miss some TP nuances in specific scenarios.

**Table 2:** Research question 1 (RQ1) model coefficients table of main effect predictors (contextual integrity parameters).

RQ1 main effect predictors (contextual integrity parameters)		Estimate β	OR^a^ (95% CI)	*P* value
**Attribute**				
	Test results	.00	1.00 (—^b^)	—
	Exposure status	.07	1.08 (0.68-1.72)	.76
	Vaccination records	–.17	0.84 (0.53-1.34)	.47
**Transmission principles**				
	Public health	.00	1.00 (—)	—
	Scenario-specific	.08	1.09 (0.73-1.62)	.69
**Recipient**				
	Essential stores	.00	1.00 (—)	—
	Dine-in locations	.28	1.32 (0.80-2.21)	.28
	Domestic flights	2.42	11.34 (6.53-19.68)	<.001
	Employers	2.39	10.94 (6.37-18.78)	<.001
	Health care providers	4.98	146.36 (78.09-274.32)	<.001
	International flights	2.77	16.03 (8.88-28.92)	<.001
	Large public venues	1.41	4.13 (2.45-6.97)	<.001
	Long-distance transportation	1.77	5.87 (3.42-10.09)	<.001
	Nonessential stores	–1.05	0.35 (0.21-0.59)	<.001
	Public transit	1.13	3.12 (1.84-5.27)	<.001

^a^OR: odds ratio.

^b^Not available for baseline values.

#### Interactions Among Contextual Integrity Parameters

Our model reveals notable interactions among contextual integrity parameters, where some combinations of 2 contextual integrity parameter values yield worth-noting results in certain special cases, as shown in Table 3.

When considering recipient and attribute values pairwise, although respondents are willing to share their health information across all attributes with health care providers, the OR dropped from 146.36 to 28.22 when the attribute is contact tracing exposure status (marginal OR 28.22, 95% CI 11.57-68.84; *P*<.001). There was a similar drop in OR when sharing contact tracing exposure status with airlines operating international flights (marginal OR 6.82, 95% CI 2.85-16.32; *P*=.01). In the free-text responses of these scenarios, most respondents expressed that sharing exposure status was useful to keep health care facilities and other passengers safe, but a few doubted its necessity at the late stage of this pandemic since “now we have vaccines.” The qualitative data indicate relatively low acceptance of sharing contact tracing exposure status via mobile apps when other effective pandemic mitigation measures are available, even with the most acceptable recipients.

The combination of attribute and TP values shows that respondents are slightly less likely to share contract tracing exposure status than test results (marginal OR 0.73, 95% CI 0.71-0.75; *P*=.01) when TP is scenario-specific purposes (eg, entering a place or using a service). Many comments pointed out the questionable accuracy of phone-based contact tracing exposure status, as one respondent wrote: “I don’t trust the standards developed to determine exposure, I don’t trust the network designed to measure exposure, and I don’t trust the data on the other end of the transaction (others I might have been exposed to).” The qualitative data further support respondents’ overall reluctance to share contact tracing data, especially for nonpublic health purposes.

**Table 3:** Research question 1 (RQ1) model coefficients table of effect modifiers (interactions).

RQ1 effector modifiers (interactions)		Estimate β	OR^a^ (95% CI)	*P* value
**Attribute**×**recipient**				
	Exposure status×dine-in locations	.18	1.21 (0.65-2.25)	.56
	Vaccination records×dine-in locations	.40	1.50 (0.80-2.79)	.21
	Exposure status×domestic flights	–.40	0.68 (0.35-1.30)	.24
	Vaccination records×domestic flights	.15	1.16 (0.61-2.21)	.64
	Exposure status×employers	–.61	0.54 (0.29-1.04)	.07
	Vaccination records×employers	.55	1.75 (0.91-3.36)	.10
	Exposure status×health care providers	–1.64	0.20 (0.10-0.40)	<.001
	Vaccination records×health care providers	–.30	0.74 (0.35-1.57)	.44
	Exposure status×international flights	–.85	0.43 (0.22-0.85)	.02
	Vaccination records×international flights	.35	1.43 (0.71-2.88)	.32
	Exposure status×large public venues	–.27	0.77 (0.41-1.46)	.42
	Vaccination records×large public venues	.01	1.01 (0.54-1.91)	.97
	Exposure status×long-distance transportation	–.58	0.56 (0.29-1.08)	.09
	Vaccination records×long-distance transportation	.12	1.13 (0.58-2.18)	.72
	Exposure status×nonessential stores	.17	1.20 (0.63-2.28)	.59
	Vaccination records×nonessential stores	.32	1.39 (0.73-2.62)	.32
	Exposure status×public transit	–.14	0.87 (0.46-1.64)	.67
	Vaccination records×public transit	.18	1.20 (0.65-2.24)	.56
**Attribute**×**TP^b^**				
	Exposure status×scenario-specific	–.39	0.68 (0.51-0.92)	.01
	Vaccination records×scenario-specific	–.29	0.75 (0.55-1.01)	.06
**TP**×**recipient**				
	Scenario-specific×dine-in locations	–.02	0.98 (0.59-1.63)	.94
	Scenario-specific×domestic flights	.02	1.02 (0.60-1.73)	.94
	Scenario-specific×employers	.06	1.07 (0.63-1.81)	.81
	Scenario-specific×health care providers	–.53	0.59 (0.33-1.07)	.08
	Scenario-specific×international flights	.54	1.72 (0.99-2.98)	.05
	Scenario-specific×large public venues	–.02	0.99 (0.59-1.67)	.97
	Scenario-specific×long-distance transportation	–.05	0.96 (0.56-1.64)	.88
	Scenario-specific×nonessential stores	.38	1.46 (0.86-2.47)	.16
	Scenario-specific×public transit	.01	1.02 (0.61-1.71)	.94

^a^OR: odds ratio.

^b^TP: transmission principle.

#### Demographics and COVID-19 Experiences

As shown in Table 4, political leaning is the only statistically significant demographic predictor. Compared to self-identified political moderates, self-identified liberals (OR 4.85, 95% CI 2.44-9.61; *P*<.001) are more likely to accept using COVID-19 mitigation apps. Age, living areas, education, and income did not significantly affect respondents’ acceptance levels. Interestingly, some prior COVID-19 experiences also turned out to be significant. Respondents who have installed COVID-19 contact tracing apps (OR 5.01, 95% CI 2.34-10.73; *P*<.001) and who have received COVID-19 vaccination (OR 10.54, 95% CI 4.92-22.56; *P*<.001) were generally more acceptable about data sharing via COVID-19 mitigation apps. These indicate that people’s familiarity with COVID-19 mitigation apps and their attitude toward vaccination may play a role in their acceptance of COVID-19 mitigation apps.

**Table 4:** Research question 1 (RQ1) model coefficients table of demographic and COVID-19 predictors.

RQ1 demographic and COVID-19 predictors		Estimate β	OR^a^ (95% CI)	*P* value
**Sex**				
	Female	.00	1.00 (—^b^)	—
	Male	–.17	0.85 (0.48-1.50)	.57
	Nonbinary	–.09	0.92 (0.12-7.06)	.94
**Age (years)**				
	60+	.00	1.00 (—)	—
	18-29	.16	1.18 (0.48-2.88)	.72
	30-39	.13	1.14 (0.47-2.81)	.77
	40-49	.55	1.75 (0.70-4.34)	.23
	50-59	–.20	0.82 (0.35-1.90)	.64
**Politics**				
	Moderate	.00	1.00 (—)	—
	Conservative	–.79	0.46 (0.20-1.03)	.06
	Liberal	1.57	4.85 (2.45-9.61)	<.001
**Living area**				
	Town or suburb	.00	1.00 (—)	—
	City	.25	1.30 (0.71-2.39)	.40
	Rural area	.44	1.55 (0.65-3.72)	.32
**Education**				
	High school	.00	1.00 (—)	—
	College	–.76	0.47 (0.21-1.08)	.07
	Grad school	–.30	0.75 (0.26-2.18)	.59
**Income (US $)**				
	50,000-99,999	.00	1.00 (—)	—
	25,000-49,999	.22	1.26 (0.59-2.67)	.55
	Less than 25,000	.52	1.68 (0.72-3.93)	.28
	More than 100,000	.64	1.91 (0.89-4.12)	.10
**COVID** **-19** **test**				
	No	.00	1.00 (—)	—
	Yes	.39	1.48 (0.82-2.66)	.19
**COVID** **-19** **app**				
	No	.00	1.00 (—)	—
	Yes	1.61	5.01 (2.34-10.73)	<.001
**COVID** **-19** **vaccination**				
	No	.00	1.00 (—)	—
	Yes	2.35	10.54 (4.93-22.55)	<.001
**COVID** **-19** **illness**				
	No	0.00	1.00 (—)	—
	Yes	.54	1.72 (0.98-3.03)	.06

^a^OR: odds ratio.

^b^Not available for baseline values.

### RQ2: Acceptance of Data Retention Through COVID-19 Mitigation Apps

#### Contextual Integrity Parameters

As shown in Table 5, we included 2 contextual integrity parameters (recipient and attribute) as predictors in the RQ2 model because data retention practices are considered TP. Recipient remains the most impactful contextual integrity parameter for respondents’ acceptance of data retention by COVID-19 mitigation apps. Compared to the baseline of essential stores, all other recipients are more accepted if they retain respondents’ health data from various COVID-19 mitigation apps. The largest effect remains in health care providers (OR 251.77, 95% CI 172.17-368.16; *P*<.001), followed by employers (OR 24.34, 95% CI 24.21-24.48; *P*<.001) and airlines operating international flights (OR 16.36, 95% CI 10.89-24.57; *P*<.001). These results resemble those of RQ1, showing that respondents’ acceptance of data retention practices primarily depends on recipients. Notably, the free-text responses reveal nuanced opinions on retention time. For example, even with the most accepted recipient health care providers, one respondent felt sharing test results was “only acceptable for the duration of the medical treatment,” and another commented on retaining exposure status: “After a point, it will become outdated and useless, and should be deleted for privacy reasons.” The qualitative data suggest that the acceptance of data retention practices is associated with the necessity of specific scenarios.

Regarding attribute, respondents expressed slightly greater acceptance of having their vaccination records (OR 1.69, 95% CI 1.68-1.70; *P*<.001) and their contact tracing exposure status (OR 1.57, 95% CI 1.56-1.58; *P*<.001) retained by recipients compared to the baseline of COVID-19 test results, which differs from RQ1 results. However, the effect is small, and there were no notable themes in the free-text responses to clearly explain such differences.

**Table 5:** Research question (RQ2) model coefficients table of main effect predictors (contextual integrity parameters).

RQ2 main effect predictors (contextual integrity parameters)		Estimate β	OR^a^ (95% CI)	*P* value
**Attribute**				
	Test results	.00	1.00 (—^b^)	—
	Exposure status	.45	1.57 (1.56-1.58)	<.001
	Vaccination records	.52	1.69 (1.68-1.70)	<.001
**Recipient**				
	Essential stores	.00	1.00 (—)	—
	Dine-in locations	.53	1.71 (1.15-2.54)	.008
	Domestic flights	2.25	9.50 (9.45-9.55)	<.001
	Employers	3.19	24.34 (24.21-24.48)	<.001
	Health care providers	5.52	251.77 (172.17-368.16)	<.001
	International flights	2.79	16.36 (10.89-24.57)	<.001
	Large public venues	1.36	3.93 (2.66-5.81)	<.001
	Long-distance transportation	2.03	7.67 (5.08-11.59)	<.001
	Nonessential stores	.17	1.20 (1.19-1.20)	<.001
	Public transit	1.78	5.96 (5.92-5.99)	<.001

^a^OR: odds ratio.

^b^Not available for baseline values.

#### Interactions Among Contextual Integrity Parameters

As shown in Table 6, many combinations of attribute and recipient values show that respondents’ acceptance of data retention through COVID-19 mitigation apps dropped significantly, demonstrating the potential inappropriateness for certain recipients to retain their phone-based contact tracing exposure status and their vaccination records.

When attribute is phone-based contact tracing exposure status, respondents’ acceptance of data retention significantly decreased for 7 of 10 recipients, with the largest drop in the most accepted recipients including health care providers (marginal OR 48.91, marginal 95% CI 29.04-82.41; *P*<.001), employers (marginal OR 6.11, marginal 95% CI 3.64-10.25; *P*<.001), airliners operating international flights (marginal OR 6.82, marginal 95% CI 3.72-11.56; *P*<.001). In the free-text responses, one respondent found it acceptable for employers to retain exposure status data when “the virus was transmittable,” while another believed exposure status “is not needed now in 2023” for air travel. There are smaller acceptance decreases for airlines operating domestic flights (marginal OR 5.47, marginal 95% CI 3.22-9.29; *P*<.001), long-distance transportation operators (marginal OR 3.53, marginal 95% CI 2.02-6.16; *P*=.008), and local public transit operators (marginal OR 3.49, marginal 95% CI 2.05-5.95; *P*<.001). Notably, the acceptance of nonessential stores retaining data (marginal OR 0.91, marginal 95% CI 0.52-1.62; *P*<.001) dropped even below that of the baseline as one respondent commented: “I don’t see the need for nonessential stores to keep my private data.” These results resonate with RQ1: people were less comfortable with data practices around phone-based contact tracing data due to lack of necessity.

When attribute is COVID-19 vaccination records, data retention acceptance decreased for 4 recipients, with large drops for local public transit operators (marginal OR 3.19, marginal 95% CI 1.89-5.38; *P*=.001), large public venues (marginal OR 2.25, marginal 95% CI 1.32-3.82; *P*=.043), and nonessential stores (marginal OR 0.64, marginal 95% CI 0.36-1.12; *P*=.005) and a small drop for employers (marginal OR 22.87, marginal 95% CI 13.80-37.92; *P*<.001). The free-text responses revealed that many respondents believed vaccination records were medical records and “shouldn’t be disclosed and even less stored or kept.” Others worried about potential data breaches after data retention that “leaked information about a person’s medical history and choices.” Another respondent found it acceptable for large public venues to keep it, but “it should be deleted after the event has ended and resubmitted for every event.” This qualitative evidence suggests the inappropriateness for some everyday services and venues to retain people’s vaccination records due to the medical nature of the data.

**Table 6:** Research question (RQ2) model coefficients table of effect modifiers (interactions).

RQ2 effect modifiers (interactions)		Estimate β	OR^a^ (95% CI)	*P* value
**Attribute**×**recipient**				
	Exposure status×dine-in locations	–.28	0.76 (0.44-1.31)	.32
	Vaccination records×dine-in locations	–.37	0.69 (0.40-1.19)	.19
	Exposure status×domestic flights	–.55	0.58 (0.58-0.58)	<.001
	Vaccination records×domestic flights	–.18	0.84 (0.58-1.21)	.35
	Exposure status×employers	–1.38	0.25 (0.25-0.25)	<.001
	Vaccination records×employers	–.06	0.95 (0.94-0.95)	<.001
	Exposure status×health care providers	–1.63	0.20 (0.12-0.33)	<.001
	Vaccination records×health care providers	–.01	1.00 (0.59-1.69)	.99
	Exposure status×international flights	–.95	0.39 (0.23-0.66)	<.001
	Vaccination records×international flights	–.35	0.71 (0.42-1.20)	.20
	Exposure status×large public venues	–.28	0.76 (0.45-1.29)	.31
	Vaccination records×large public venues	–.55	0.58 (0.34-0.98)	.04
	Exposure status×long-distance transportation	–.77	0.47 (0.27-0.82)	.008
	Vaccination records×long-distance transportation	–.34	0.71 (0.41-1.24)	.23
	Exposure status×nonessential stores	–.26	0.77 (0.77-0.78)	<.001
	Vaccination records×nonessential stores	–.62	0.54 (0.35-0.83)	.005
	Exposure status×public transit	–.53	0.59 (0.59-0.59)	<.001
	Vaccination records×public transit	–.62	0.54 (0.37-0.79)	.001

^a^OR: odds ratio.

#### Demographics and COVID-19 Experiences

As shown in Table 7, several demographic predictors have significant effects in the RQ2 model. Similar to RQ1, self-identified liberals (OR 2.03, 95% CI 2.01-2.04; *P*<.001) are slightly more likely to accept recipients to retain their health data via a mobile app. We also observe a significant but small effect for respondents aged 30 to 39 years (OR 1.03, 95% CI 1.02-1.03; *P*<.001) compared to those aged 60 years and older. In the opposite direction, compared to those with high school education, respondents with college education are less acceptable toward data retention practices (OR 0.77, 95% CI 0.77-0.78; *P*<.001). Similarly, we found a significant but smaller effect for respondents with self-reported annual income of US $25,000 to US $49,999 (OR 0.99, 95% CI 0.99-1.00; *P*=.003) compared to those with self-reported annual income of US $50,000 to US $99,999. Regarding COVID-19 experiences, respondents who have installed COVID-19 contact tracing apps (OR 3.39, 95% CI 1.77-6.48; *P*<.001), who have received COVID-19 vaccination (OR 4.74, 95% CI 4.71-4.77; *P*<.001), and who knew someone who got seriously ill or passed away (OR 1.84, 95% CI 1.16-2.90; *P*=.009) are more likely to accept data retention practices by various recipients.

**Table 7:** Research question (RQ2) model coefficients table of demographic and COVID-19 predictors.

RQ2 demographic and COVID-19 predictors		Estimate β	OR^a^ (95% CI)	*P* value
**Sex**				
	Female	.00	1.00 (—^b^)	—
	Male	.15	1.17 (0.72-1.89)	.53
	Nonbinary	.64	1.91 (0.33-11.04)	.47
**Age (years)**				
	60+	.00	1.00 (—)	—
	18-29	.60	1.82 (0.95-3.50)	.07
	30-39	.02	1.03 (1.02-1.03)	<.001
	40-49	.50	1.65 (0.82-3.32)	.16
	50-59	–.18	0.84 (0.44-1.57)	.58
**Politics**				
	Moderate	.00	1.00 (—)	—
	Conservative	–.50	0.61 (0.33-1.12)	.11
	Liberal	.70	2.03 (2.01-2.04)	<.001
**Living**				
	Town or suburb	.00	1.00 (—)	—
	City	–.10	0.91 (0.54-1.52)	.71
	Rural area	.05	1.06 (0.50-2.25)	.88
**Education**				
	High school	.00	1.00 (—)	—
	College	–.26	0.77 (0.77-0.78)	<.001
	Grad school	.16	1.18 (0.60-2.29)	.64
**Income (US $)**				
	50,000-99,999	.00	1.00 (—)	—
	25,000-49,999	–.01	0.99 (0.99-1.00)	.003
	Less than 25,000	–.15	0.87 (0.46-1.63)	.66
	More than 100,000	–.02	0.98 (0.54-1.80)	.96
**COVID** **-19** **test**				
	No	.00	1.00 (—)	—
	Yes	.14	1.16 (0.70-1.91)	.57
**COVID** **-19** **app**				
	No	.00	1.00 (—)	—
	Yes	1.22	3.39 (1.77-6.48)	<.001
**COVID** **-19** **vaccination**				
	No	.00	1.00 (—)	—
	Yes	1.55	4.74 (4.71-4.77)	<.001
**COVID** **-19** **illness**				
	No	.00	1.00 (—)	—
	Yes	.60	1.84 (1.16-2.90)	.009

^a^OR: odds ratio.

^b^Not available for baseline values.

### Summary of Results

For RQ1, we discovered that the contextual integrity parameter recipient has the most significant effect on how willing respondents are to share data through COVID-19 mitigation apps and that certain combinations of recipient, attribute, and TP values also influence their willingness. These results, taken together, reject NH1.1. Among demographic variables, self-reported political leaning and prior COVID-19 experiences impact respondents’ overall acceptance, rejecting NH1.2.

For RQ2, the contextual integrity parameter recipient greatly influences respondents’ acceptance of data retention practices via COVID-19 mitigation apps. The contextual integrity parameter attributes also turned out to be significant, as respondents felt that retaining their COVID-19 test results was less acceptable. Moreover, many combinations of attribute and recipient values further impact respondents’ acceptance of data retention practices via COVID-19 mitigation apps. These results, taken together, reject NH2.1. Regarding demographics, besides respondents’ political leaning and prior COVID-19 experiences as in RQ1 results, age, self-reported education, and annual income also have significant effects on respondents’ acceptance of data retention, thereby rejecting NH2.2.

## Discussion

### Contributions of This Study

By surveying a US representative sample, this study confirms prior research that data privacy is a key factor impacting the adoption of COVID-19 mitigation apps [3,6,7,15,19,20] and that the specific contexts impact people’s privacy attitudes toward these apps [15,31,34]. Different from prior studies that only examined user-centered data privacy for one type of COVID-19 mitigation apps [3,6,7,21,22], our study revealed US respondents’ varying acceptance of different types of COVID-19 mitigation apps that collect different types of personal health data. This highlights the importance of holistically examining multiple COVID-19 mitigation apps after the pandemic dust settles, providing insights into the deployment of different types of pandemic mitigation apps to combat future public health crises.

Besides exploring the general acceptance of sharing personal health data via COVID-19 mitigation apps, this study dived into people’s attitudes toward data retention practices via these apps—a critical consideration in privacy research [23-25]. In many scenarios, we found US respondents’ acceptance differed from their acceptance of initial data sharing, highlighting the importance of thoroughly considering the data retention practices of public health mobile apps.

Finally, this study contributes to the broader application of the contextual integrity privacy framework to analyze how contextual data privacy impacts the acceptance of COVID-19 mitigation apps, showing the framework’s suitability to gauge people’s acceptance of pandemic mitigation apps and other public health technologies.

### Limitations and Future Research

We acknowledge several limitations of the study and suggest future research directions. First, this study inherits the limitations of the web-based survey methodology, where we relied on respondents’ self-reported data. We mitigated this by focusing on respondents’ self-reported attitudes rather than using attitudes to predict actual behaviors. Future research could explore data on people’s actual behaviors with COVID-19 mitigation apps to triangulate our study findings.

Second, we minimized the survey sampling bias by recruiting a US representative sample on Prolific. Still, our findings cannot fully represent the US population’s perspective or generalize beyond the United States. To provide comparative analyses, we encourage researchers to replicate our contextual integrity–based survey methods to examine people’s acceptance of COVID-19 mitigation apps in other countries or regions. This will elucidate potentially different contextual integrity information norms regarding pandemic mitigation apps across cultures.

Third, though this survey depicts US respondents’ contextual acceptance of various COVID-19 mitigation apps at the end of the pandemic, it does not show longitudinal trends about people’s privacy attitudes toward these apps. However, researchers can quickly adjust and deploy the contextual integrity–based survey methods developed in this study longitudinally, should future public health research needs arise. This way, future research could generate longitudinal insights into the acceptance of public health technologies using a consistent contextual integrity–based survey instrument.

Furthermore, we only evaluated the most important set of contextual factors according to the contextual integrity framework and the app types being examined, due to the quantitative nature of the survey and the goal of generating meaningful statistical results. In addition, though the qualitative data collected from the free-text questions enhanced our statistical results, the short survey completion time limited the depth of such data. We encourage researchers to explore a richer set of contextual factors and consider alternative research methods to generate deeper qualitative findings.

### Deployment Strategies for Future Pandemic Mitigation Apps

This study provides a cross-sectional overview of people’s contextual acceptance of 3 major types of COVID-19 mitigation apps after the pandemic in the United States, which informs the deployment strategies for future pandemic mitigation apps. Our results backed up prior contextual integrity–inspired studies [15,31,34] that contextual integrity parameters, especially recipient, attribute, and TP, can predict people’s acceptance of COVID-19 mitigation apps in different situations. This survey’s fully factorial design enabled us to extend these prior studies by investigating interactions among contextual integrity parameters. We found the recipient to be the most influential contextual integrity parameter, while the interplay among multiple contextual integrity parameters in specific scenarios complicated people’s acceptance levels. This implies that there are no one-size-fits-all data privacy norms for COVID-19 mitigation apps and that the specific contexts for data practices, often determined by a combination of contextual integrity parameters, matter. In the case of a future pandemic, we recommend public health policy makers proactively collect evidence-based data about the general public’s contextual acceptance of using various pandemic mitigation apps to inform their decisions on when, where, and how to deploy these apps.

Our statistical models for both RQs revealed that political leaning and prior COVID-19 experiences also influenced people’s acceptance of COVID-19 mitigation apps. Specifically, respondents who self-identified as liberals, have downloaded COVID-19 mitigation apps, and have been vaccinated against COVID-19 generally reported higher acceptance levels. The RQ2 model also yielded a few significant demographic predictors (eg, age, income, and education) with smaller effects. These results suggest that public health policy makers must consider how population demographics could affect the deployment of future pandemic mitigation apps. Additionally, other public health efforts, such as general education on vaccination and publicity for public health technologies, may boost the overall acceptance of future pandemic mitigation apps.

### Implications for Future Public Health Mobile Apps

By articulating the contextual acceptance of various COVID-19 mitigation apps deployed in this pandemic, this study yielded rich implications for future public health mobile apps. Built upon prior studies focusing on one type of COVID-19 mitigation app [3,6,7,21,22], this study compared 3 types of apps (ie, contact tracing, test result reporting, and proof of vaccination) that have long-term utility in future public health crises. Our results suggested that people were least comfortable with apps that perform phone-based contact tracing, contrasting the relatively high acceptance and adoption of contact tracing apps early in the pandemic [3]. One explanation is that, as more effective mitigation tools (such as vaccination) become widely available in the United States near the end of the pandemic, people may not perceive phone-based contact tracing as necessary or appropriate, especially given its data privacy implications. This highlights the need for policy makers to strategize what public health mobile apps to promote according to the changing pandemic stages and available mitigation methods.

Our findings also shed light on the nuanced implications of the data retention practices of COVID-19 mitigation apps. This study provided initial evidence that people’s acceptance of data retention diverged from their acceptance of initial data sharing in specific scenarios, where many respondents felt data should not be retained indefinitely even for the most accepted recipients (eg, health care providers). As we are phasing out the COVID-19 mitigation apps, it is critical to re-evaluate how these apps retain data collected during the pandemic to minimize downstream privacy harms, such as breaches of retained data.

Last but not least, our findings informed how to appropriately deploy future public health mobile apps in the face of emergencies and crises. The different acceptance levels across our vignette scenarios suggest the importance of considering people’s contextual acceptance of public health apps’ data practices. Deployment of public health apps should start with necessary scenarios that the general public finds acceptable and avoid controversial scenarios.

### Conclusions

This study systematically applies the contextual integrity framework to examine people’s contextual data privacy attitudes toward multiple types of COVID-19 mitigation apps that emerged during the COVID-19 pandemic. It confirmed that contextual integrity parameters can help predict people’s acceptance of using these apps in various realistic scenarios, yield novel evidence on the acceptance of data retention practices by these apps, and generate rich implications for deploying future public health mobile apps.

## Appendix

Multimedia Appendix 1 Demographic distributions of the survey sample.

## Abbreviations

NH: null hypothesis
OR: odds ratio
RQ: research question
TP: transmission principle
