# Supplementary material for: Contextual Acceptance of COVID-19 Mitigation Mobile Apps in the United States: Mixed Methods Survey Study on Postpandemic Data Privacy
Source: J Med Internet Res. 2024 Aug 29;26:e57309. doi: 10.2196/57309 (PMC11393507; doi:10.2196/57309)
Supplement: Multimedia Appendix 1 [file jmir_v26i1e57309_app1.docx]

The study sample’s demographic distributions compared to recent estimates of U.S. population demographic distributions, demonstrating the representativeness of our sample.

| value | total | study % | US population % |
| --- | --- | --- | --- |
| Age | | | |
| 18-29 | 148 | 22.0% | 20.6% |
| 30-39 | 125 | 18.5% | 17.4% |
| 40-49 | 110 | 16.3% | 15.8% |
| 50-59 | 137 | 20.3% | 16.0% |
| 60+ | 154 | 22.8% | 30.3% |
| Education | | | |
| High school | 110 | 16.3% | 27.7% |
| College | 452 | 67.1% | 58.0% |
| Grad School | 111 | 16.5% | 14.3% |
| I prefer not to answer | 1 | 0.1% | 0.0% |
| Income | | | |
| Less than $25,000 | 132 | 19.6% | 32.3% |
| $25,000 - $49,999 | 170 | 25.2% | 26.2% |
| $50,000 - $99,999 | 206 | 30.6% | 26.4% |
| More than $100,000 | 151 | 22.4% | 15.1% |
| I prefer not to answer | 15 | 2.2% | 0.0% |
| Location | | | |
| Town or suburb | 312 | 46.3% | 31.0% |
| City | 271 | 40.2% | 55.0% |
| Rural area | 91 | 13.5% | 14.0% |
| Politics | | | |
| Moderate | 184 | 27.3% | 37.0% |
| Conservative | 155 | 23.0% | 36.0% |
| Liberal | 335 | 49.7% | 25.0% |
| Gender | | | |
| Female | 340 | 50.4% | 49.9% |
| Male | 317 | 47.0% | 47.7% |
| Non-binary | 14 | 2.1% | 2.4% |
| Prefer not to answer | 3 | 0.4% | 0.0% |
| Race | | | |
| African American | 85 | 12.6% | 13.6% |
| Asian | 40 | 5.9% | 6.3% |
| Caucasian | 495 | 73.4% | 58.9% |
| Hispanic/Latinx | 37 | 5.5% | 19.1% |
| Native American | 2 | 0.3% | 1.3% |
| Other/unknown | 1 | 0.1% | 0.0% |
| Two or more of the above | 10 | 1.5% | 3.0% |
| I prefer not to answer | 4 | 0.6% | 0.0% |

**Appendix references:**

U.S. Census Bureau. 2022 Census Quick Facts. https://www.census.gov/quickfacts/fact/table/US/PST045222. [accessed on November 20, 2023]

Gallup. Democrats’ Identification as Liberal Now 54%, a New High. 12 Jan. 2023. https://news.gallup.com/poll/467888/democrats-identification-liberal-new-high.aspx. [accessed on November 20, 2023]

Parker K., Horowitz JM, Brown A, Fry R, Cohn D, Igielnik R. Pew Research Center. What Unites and Divides Urban, Suburban and Rural Communities. Pew Research Center’s Social & Demographic Trends Project. 22 May 2018. https://www.pewresearch.org/social-trends/2018/05/22/what-unites-and-divides-urban-suburban-and-rural-communities/. [accessed on November 20, 2023]

U.S. Census Bureau. Sexual Orientation and Gender Identity in the Household Pulse Survey. https://www.census.gov/library/visualizations/interactive/sexual-orientation-and-gender-identity.html. [accessed on November 20, 2023]

U.S. Census Bureau. Age Heaping in the 2020 Census Demographic and Housing Characteristics File (DHC). https://www.census.gov/newsroom/blogs/random-samplings/2023/05/age-heaping-2020-census-dhc.html. [accessed on November 20, 2023]

U.S. Census Bureau. Educational Attainment in the United States: 2022. https://www.census.gov/data/tables/2022/demo/educational-attainment/cps-detailed-tables.html. [accessed on November 20, 2023]

U.S. Census Bureau. PINC-01. Selected Characteristics of People 15 Years and Over, by Total Money Income, Work Experience, Race, Hispanic Origin, and Sex. https://www.census.gov/data/tables/time-series/demo/income-pov [accessed on November 20, 2023]
